# Hypertension among persons living with HIV/AIDS and its association with HIV-related health factors

**DOI:** 10.1186/s12981-023-00576-2

**Published:** 2024-01-11

**Authors:** Mawulorm K. I. Denu, Ritika Revoori, Maame Araba E. Buadu, Oluwakemi Oladele, Kofi Poku Berko

**Affiliations:** 1 Department of Medicine, University of Massachusetts Chan Medical School, Worcester, MA 01655 USA; 2 Department of Medicine, Massachusetts General Hospital, Boston, MA USA; 3 Department of Psychiatry, Korle Bu Teaching Hospital, Accra, Ghana; 4 Infectious Disease Unit, Korle Bu Teaching Hospital, Accra, Ghana

**Keywords:** HIV, Persons living with HIV (PLHIV), Hypertension, Antiretroviral medications

## Abstract

**Background:**

Human Immunodeficiency Virus **(**HIV) infection remains a public health concern in many countries. The increased life expectancy in the post-Antiretroviral Therapy (ART) era has led to an increased risk of cardiovascular disease and death among Persons Living with HIV (PLHIV). Hypertension remains a significant risk factor for cardiovascular disease among PLHIV. Some studies have suggested associations between hypertension among PLHIV and HIV-related health factors.

**Objective:**

To determine the prevalence of hypertension among PLHIV on antiretroviral medications and examine its association with HIV-related health factors.

**Methods:**

A cross-sectional study was conducted among attendants at an adult HIV clinic. 362 study participants were selected by systematic sampling. Data on hypertension diagnosis, HIV-related health factors, sociodemographic and other traditional cardiovascular risk factors were collected using a standardized questionnaire and patient chart review. Multivariate logistic regression model was used to determine the association between hypertension and HIV-related factors, adjusting for other risk factors for hypertension.

**Results:**

The mean age of participants was 47.9 years and majority of participants were female (77.1%). 42% of study participants had been on antiretroviral medications for > 10 years. The prevalence of hypertension was 17.4%. Age > 50 years was associated with higher odds of hypertension (aOR: 3.75, 95%CI 1.68, 8.55, p-value: 0.002). BMI in overweight and obese categories, and a history of comorbid medical conditions (diabetes, hyperlipidemia) were also associated with higher odds of hypertension (aOR: 3. 76, 95%CI 1.44, 9.81, p-value: 0.007), (aOR: 3.17, 95%CI 1.21, 8.32, p-value: 0.019) and (aOR: 14.25, 95%CI 7.41, 27.41, p-value: < 0.001) respectively. No HIV-related health factors were associated with hypertension.

**Conclusion:**

Hypertension was a common condition among PLHIV on antiretroviral medications. No HIV-related health factors were associated with hypertension. Traditional risk factors associated with hypertension were increased age > 50 years, increased BMI, and a history of comorbid medical conditions.

## Background

HIV continues to pose a significant public health challenge worldwide, with more than 37 million individuals living with the virus [1]. Although antiretroviral medications have markedly reduced mortality rates, a cure for HIV remains elusive [2]. Nevertheless, the incidence of cardiovascular disease-related mortality has notably risen among People Living with HIV (PLHIV) [3, 4]. Hypertension, a well-established risk factor for cardiovascular disease and mortality [5–7], is emerging as a growing concern among this population [3, 8]. Sub-Saharan African countries, which bear the highest global burden of both HIV and hypertension, are particularly affected [1, 9].

The prevalence of hypertension among PLHIV has surged, largely attributable to increased life expectancy resulting from expanded access to antiretroviral medications [10, 11]. A study conducted in Kenya estimated the prevalence of hypertension among PLHIV on antiretroviral therapy to range from 6 to 50% [12]. Similarly, investigations in Burundi and Zambia reported prevalence rates of 17.4% and 18.4%, respectively [13, 14].

Various mechanisms have been proposed to explain the heightened risk of hypertension in HIV-infected individuals, including microbial translocation, chronic inflammation, immune reconstitution, lipodystrophy, adipocytokines, neuroendocrine responses, and HIV-related renal disease [15–20]. Epidemiological factors contributing to the increased prevalence of hypertension among PLHIV are manifold. The improved survival of PLHIV receiving antiretroviral therapy is associated with a heightened risk of developing traditional hypertension risk factors, such as advancing age, lifestyle-related risk factors, and comorbid medical conditions [13, 21, 22].

Certain HIV-related health factors have also been linked to hypertension among PLHIV. Some studies have suggested that the use of specific antiretroviral medications, notably those in the protease inhibitors (PIs) and integrase strand transfer inhibitors (INSTI) classes, may increase the incidence of metabolic syndrome and weight gain, thereby elevating the risk of hypertension [12, 14, 23, 24]. Furthermore, a study conducted in Ethiopia identified associations between hypertension among PLHIV and the duration of HIV infection and the duration of antiretroviral medication use [22]. While certain studies have suggested a connection between CD4 count and hypertension, others have not found a significant association [3, 7, 25].

Given the substantial burden of both HIV and hypertension in Sub-Saharan Africa, there is a pressing need for further research examining the interplay between these two conditions in this region. Consequently, our study aims to determine the prevalence of hypertension among PLHIV at the Infectious Disease Unit of the Korle-Bu Teaching Hospital in Ghana, while also exploring its association with some HIV-related health factors.

## Materials and methods

### Study design and data collection

This was a facility-based analytic cross-sectional study among attendants of the adult HIV clinic at Korle Bu Teaching Hospital between June and August 2020. The study was limited to PLHIV aged 18 years and above who had been on antiretroviral treatment for at least 6 months. A standardized questionnaire was used for data collection on hypertension diagnosis, sociodemographic and health factors of study participants. In addition, participant health information such as confirmation of hypertension diagnosis, BMI, and comorbid medical conditions as well as HIV-related health factors including duration of HIV diagnosis, duration on antiretrovirals, HIV-typing, ART regimen, and viral load within the past year was extracted from participants’ medical records.

Self-adherence to medications was ascertained for all participants. The study protocol (KBTH-IRB/00047/2020) was reviewed and approved by the Korle Bu Teaching Hospital Institutional Review Board on 27th May 2020.

### Dependent and explanatory variables

The dependent variable of interest was a diagnosis of hypertension. This was derived from participants' responses of ‘Yes’ or ‘No’ to a history of hypertension. Diagnosis was confirmed with a review of participants’ medical records.

### HIV-related health factors

Health-related factors examined in this study were HIV type, HIV viral load, duration of HIV diagnosis, duration of ART treatment, ART regimen, ART adherence rate, and comorbidities. These were obtained from patients using the questionnaire and review of patient health records.

ART adherence rate was assessed by a standardized 30 day recall of medications using the Brief Adherence Rating Scale (BARS) and pill count. History of comorbid medical conditions (diabetes and hyperlipidemia), smoking history and BMI were also derived from medical record reviews.

### Sociodemographic factors

Data were collected on sociodemographic factors such as age, education, sex, marital status, using a standardized questionnaire.

### Statistical analyses

Descriptive statistics were first produced for each variable. Following this, univariate tests were performed to examine the association between hypertension and HIV-related health factors, socio-demographic factors and some traditional risk factors of hypertension.

Multiple logistic regression was then utilized to examine the association between hypertension and HIV-related health factors adjusting for sociodemographic and other predictor variables. All analyses were done with STATA (version 16). Statistical tests were 2-sided, and the significance level was set at 0.05.

## Results

Mean age was 47.9 years and majority of participants were female (77.1%). 42% of study participants had been on antiretroviral medications for > 10 years. The prevalence of hypertension was 17.4% (63/362). Univariate analysis showed no difference in HIV-related health factors between participants who had hypertension and those without hypertension.

Age, history of comorbid medical conditions (diabetes, hyperlipidemia), BMI category were statistically associated with hypertension. Table 1 summarizes the results of univariate tests.

**Table 1:** Characteristics of PLHIV by hypertension status

Variables	Hypertension		p-value
	Yes (n = 63)	No (n = 299)	
Gender			**0.883**
Male	14 (22.2%)	69 (23.1%)	
Female	49 (77.8%)	230 (76.9%)	
Age			** < 0.001**
< 50 years	20 (31.8%)	183 (61.2%)	
50 years	43 (68.2%)	116 (38.8%)	
Education level			**0.430**
Less than high school	29 (46%)	154 (51.5%)	
High school and above	34 (54%)	145 (48.5%)	
Marital status			**0.424**
Married	28 (44.4%)	121 (40.5%)	
Single	25 (39.7)	108 (36.1%)	
Divorced/separated	10 (15.9%)	70 (23.4%)	
History of comorbid medical condition			** < 0.001**
Yes	63 (100%)	29 (9.7%)	
No	0 (0%)	270 (90.3%)	
Smoking status			**0.176**
Smoker	19 (30.2%)	64 (21.4%)	
Non-smoker	44 (69.8%)	235 (78.6%)	
BMI category			**0.004**
Underweight	3 (4.8%)	17 (5.7%)	
Normal	20 (31.8%)	156 (52.2%)	
Overweight	19 (30.1%)	79 (26.4%)	
Obese	21 (33.3%)	47 (15.7%)	
HIV type			**0.567**
Type 1	61 (96.8%)	193 (98%)	
Type 2	2 (3.2%)	6 (2%)	
Duration of HIV diagnosis			**0.521**
< 2 years	2 (3.2%)	24 (8%)	
2–5 years	9 (14.3%)	50(16.7%)	
5–10 years	22 (38.1%)	104 (34.8%)	
> 10 years	28 (44.4%)	121 (40.5%)	
Duration on ART			**0.319**
< 10 years	33 (52.4%)	177 (59.2%)	
10 years	30 (47.6%)	122 (40.8%)	
HIV viral load			**0.667**
$\le$1000 cp/ml	59 (93.7%)	284(95%)	
> 1000 cp/ml	4 (6.4%)	15(5%)	
ART adherence rate			**0.456**
95%	16 (25.4%)	90 (30.1%)	
> 95%	47 (74.6%)	209 (69.9%)	
ART regimen			**0.715**
NNRTI-based	47 (74.6%)	234 (78.3%)	
INSTI-based	13 (20.6%)	49 (16.4%)	
PI-based	3 (4.8%)	16 (5.3%)	

p-values for differences in baseline characteristics between the 2 groups by hypertension status

Results of the multivariate logistic regression showed that adjusted for socio-demographic factors and traditional risk factors for hypertension, no HIV-related health factor was associated with hypertension. Table 2 provides a summary of results of the multivariate logistic regression.

**Table 2:** Multivariate logistic regression analysis of the factors associated with hypertension among PLHIV

Variable	aOR (95%CI)	SE	p-value
Sex			
Female	Ref.		
Male	0.56 (0.21,1.51)	0.28	0.252
Age category			
< 50 years	Ref.		
50 years	3.75 (1.65,8.55)	1.57	0.002
Marital status			
Married	Ref.		
Single	1.52 (0.51,4.49)	0.84	0.453
Divorced/Separated	1.37 (0.46,4.10)	0.57	0.568
Education			
Less than high school	Ref.		
High school and above	0.69 (0.32,1.46)	0.27	0.334
Smoking			
Non-smoker	Ref.		
Smoker	1.45 (0.61,3.46)	0.64	0.396
BMI category			
Normal	Ref.		
Underweight	2.07 (0.37,11.66)	1.83	0.408
Overweight	3.76 (1.44,9.81)	1.84	0.007
Obese	3.17 (1.21,8.32)	1.56	0.019
Comorbid medical condition			
No	Ref.		
Yes	14.25 (7.41,27.41)	4.75	< 0.001
HIV type			
Type 1	Ref.		
Type 2	0.12 (0.01,2.03)	0.17	0.140
Duration of HIV diagnosis			
< 2 years	Ref.		
2–5 years	6.69 (0.22,20.19)	11.72	0.278
5–10 years	3.73 (0.13,10.88)	6.33	0.437
> 10 years	0.38 (0.01,32.24)	0.87	0.672
Duration on ART			
< 10 years	Ref.		
> 10 years	0.11 (0.01,2.04)	1.17	0.141
HIV viral load			
1000 cp/ml	Ref.		
> 1000 cp/ml	0.43 (0.07,2.47)	0.38	0.347
ART adherence rate			
95%	Ref.		
> 95%	1.99 (0.75,5.32)	0.99	0.167
ART regimen			
NNRTI-based	Ref.		
INSTI-based	1.04 (0.39,2.83)	0.53	0.932
PI-based	0.58 (0.07,4.52)	0.61	0.602

## Discussion

From our study, we observed a prevalence of hypertension among People Living with HIV (PLHIV) at 17.4%. This prevalence aligns closely with findings from studies conducted in Burundi and Zambia [13, 14]. Slightly lower prevalence rates were reported from studies conducted in Ethiopia (14%) and Tanzania (8%) [26, 27]. Conversely, Dzudie et al. reported a higher rate of 23.9% in Cameroon [28], and a separate study from Nigeria found a prevalence rate of 24.9% [29]. Other studies reported hypertension prevalence rates among PLHIV to be 27% and 53% in Uganda and South Africa, respectively [30, 31]. Within the general population of Ghana, hypertension prevalence has been reported to range between 13% and 27.3% [32, 33]. These findings suggest that the prevalence of hypertension among PLHIV in Ghana is in line with that observed in PLHIV populations in other Sub-Saharan African nations, and the general population in Ghana. A systematic review study estimated the global prevalence of hypertension among PLHIV to be approximately 25.2%, with studies from the USA and Malaysia reporting higher prevalence rates compared to our study’s results [34–36].

In our analysis, factors significantly associated with hypertension in our sample population included age 50 years and above, Body Mass Index (BMI) falling within the overweight and obese categories, and a history of comorbid medical conditions such as diabetes and hyperlipidemia. These risk factors have long been established as traditional associations with hypertension and cardiovascular disease [37–41]. A hypertension awareness and screening study conducted in Ghana also identified increasing age and elevated BMI as significant factors associated with hypertension in the general population [36].

Of note is the finding that factors such as the duration of HIV diagnosis, HIV type, viral load, duration on antiretroviral treatment, antiretroviral adherence rate, and antiretroviral regimen were not significantly associated with hypertension in our study. Similar findings have been reported in several studies investigating the association between hypertension and these HIV-related health factors [13, 42]. In contrast, some studies have reported associations between hypertension and antiretroviral medication regimens based on Non-Nucleoside Reverse Transcriptase Inhibitors (NNRTIs) and Protease Inhibitors (PIs) [14, 43]. Given the concerns surrounding incident hypertension attributed to antiretroviral medications among PLHIV, this finding carries important implications for clinicians, policymakers, and other stakeholders involved in HIV control programs in Ghana. However, it is important to acknowledge that our study's cross-sectional design leaves room for residual confounding, and findings may differ in studies employing alternative designs, such as cohort studies or randomized control studies(RCTs). As such, this finding does not negate the need for ongoing monitoring of PLHIV on antiretroviral medications and the implementation of hypertension screening protocols in facilities providing care to PLHIV.

In light of these findings, it is imperative to consider the adoption and implementation of integrated chronic disease management models, as demonstrated in Uganda and South Africa, which have yielded positive results in the diagnosis and management of hypertension among PLHIV [44, 45]. Sensitization of care providers responsible for PLHIV to the increasing burden of hypertension and other cardiovascular disease risk factors is also crucial in reducing cardiovascular disease-related mortality.

## Limitations

Our study has some limitations. Firstly, this was a single-site study with a cross-sectional design which lacks the ability to establish causality. The study was limited to the HIV clinic attendants who were on antiretroviral treatments, and findings may not be the same among PLHIV who are not clinic attendants or are not on antiretroviral treatment. The comorbid medical conditions used as part of the study were diabetes and dyslipidemia. This fails to consider other medical conditions that are associated with hypertension.

## Conclusion

Hypertension remains highly prevalent among PLHIV although no HIV-related health factor was associated with hypertension in this study. Hypertension was however associated with some traditional risk factors for hypertension. Given the significant prevalence of hypertension among PLHIV, there is a need for an integrative disease management approach in managing both diseases. Further studies among PLHIV on antiretroviral medication using a prospective cohort study design is needed to further study the association between hypertension and HIV-related health factors.

## Data Availability

The datasets used and analyzed during the current study are available from the corresponding author upon reasonable request.

## Abbreviations

HIV: Human Immunodeficiency Virus
PLHIV: Persons living with HIV
CVD: Cardiovascular disease
ART: Antiretroviral therapy
